# Impact of neoadjuvant FLOT treatment of advanced gastric and gastroesophageal junction cancer following surgical therapy

**DOI:** 10.3389/fsurg.2023.1148984

**Published:** 2023-04-03

**Authors:** Attila Paszt, Zsolt Simonka, Krisztina Budai, Zoltan Horvath, Marton Erdos, Marton Vas, Aurel Ottlakan, Tibor Nyari, Zoltan Szepes, Gabriella Uhercsak, Aniko Maraz, Laszlo Torday, Laszlo Tiszlavicz, Judit Olah, Gyorgy Lazar

**Affiliations:** ^1^ Department of Surgery, University of Szeged, Szeged, Hungary; ^2^ Department of Medical Physics and Informatics, University of Szeged, Szeged, Hungary; ^3^ 1st Department of Internal Medicine, University of Szeged, Szeged, Hungary; ^4^ Department of Oncotherapy, University of Szeged, Szeged, Hungary; ^5^ Department of Pathology, University of Szeged, Szeged, Hungary

**Keywords:** FLOT therapy, neoadjuvant treatment, advanced gastric tumour, gastroesophageal junction, surgery

## Abstract

**Introduction:**

Therapeutic treatment for advanced-stage (T_2_–T_4_) gastroesophageal junction (GEJ) and gastric cancer involves neoadjuvant chemotherapy with subsequent surgical intervention.

**Method:**

Neoadjuvant oncological treatment for GEJ and gastric cancer previously consisted of the intravenous administration of epirubicin, cisplatin and fluorouracil (ECF) or epirubicin, cisplatin and capecitabine (ECX) combination (Group 1). The new protocol (FLOT, F: 5-FU, L: leucovorin, O: oxaliplatin, T: docetaxel), included patients with resectable GEJ and gastric cancer who had a clinical stage cT_2_ or higher nodal positive cN+ disease (Group 2). Between 31 December 2008 and 31 October 2022, the effect of different oncological protocols in terms of surgical outcomes in cases of T_2_–T_4_ tumours were retrospectively evaluated. Results of randomly assigned patients from the earlier ECF/ECX protocol (*n* = 36) (Group 1) and the new FLOT protocol (*n* = 52) (Group 2) were compared. Effect of different neoadjuvant therapies on tumour regression, types of possible side effects, type of surgery, and oncological radicality of surgical procedures were analysed.

**Results:**

When comparing the two groups, we found that in case of the FLOT neoadjuvant chemotherapy (Group 2, *n* = 52), complete regression was achieved in 13.95% of patients, whereas in the case of ECF/ECX (Group 1, *n* = 36), complete regression occurred in only 9.10% of patients. Furthermore, in the FLOT group, the mean number of lymph nodes removed was slightly higher (24.69 vs. 20.13 in the ECF/ECX group). In terms of the safety resection margin (proximal), no significant difference was found between the two treatment groups. Nausea and vomiting were the most common side effects. The occurrence of diarrhea was significantly higher in the FLOT group (*p* = 0.006). Leukopenia and nausea occurred more commonly with the old protocol (Group 1). The rate of neutropenia was lower following FLOT treatment (*p* = 0.294), with the lack of grade II and III cases. Anaemia occured at a significantly higher rate (*p* = 0.036) after the ECF/ECX protocol.

**Conclusions:**

As a result of the FLOT neoadjuvant oncological protocol for advanced gastro-esophageal junction and gastric cancer, the rate of complete tumour regression increased significantly. The rate of side effects was also appreciably lower following the FLOT protocol. These results strongly suggest a significant advantage of the FLOT neoadjuvant treatment used before surgery.

## Introduction

The incidence of GEJ or gastric cancers vary with different geographic locations. Based on a GLOBOCAN 2020 database, gastric cancer is the fourth leading cause of cancer related deaths. In 2020 about 1,1 M newly diagnosed cases were registered worldwide (1, 2).

The rates of primary esophageal adenocarcinoma and tumours of the gastroesophageal junction (Barrett's adenocarcinoma) are constantly on the rise (3, 4). As opposed to the Siewert–Stein topographical classification (I to III) used previously (5–7), the AJCC Cancer Staging Manual (8th edition TNM) classifies cardia tumours into two large groups based on their behaviour and management (8). The first group consists of patients previously included in the Siewert I and II classes, and their treatment should follow the principles to be used in patients with esophageal tumours. Patients in the prior Siewert III class now belong to the second group, where treatment used in gastric tumour patients should be employed.

Neoadjuvant oncological treatments have been used routinely around the world for several decades now. The first treatments were developed specifically so that tumours in an inoperable stage can be subjected to surgery after a favourable response (7). Treatments with modified indications were introduced later. In these cases, the objective was not only to achieve operability but also to preserve organs and achieve better oncological results (9, 10).

Many questions have arisen during the evolution of neoadjuvant treatments (11, 12). What should the indications be exactly, what should the treatment consist of, when should restaging assessments be performed, and what is the best time of surgery (6)? In this study, we evaluated the change in the chemotherapy component of the neoadjuvant therapy. Previous treatment with 3 cycles of ECF/ECX (epirubicin, cisplatin and fluorouracil [ECF], or epirubicin, cisplatin and capecitabine [ECX]) was replaced by 4 cycles of FLOT (5-FU, leucovorin, oxaliplatin, docetaxel) (13–15).

At our department, in accordance with the protocols used previously, neoadjuvant oncological treatment is indicated for stage T2–4 advanced gastric and cardia tumours, because of the size of the tumour, local spreading and/or lymph node involvement. This therapy has numerous advantages over the adjuvant treatment administered later. It has been demonstrated to decrease the size of the tumour (downsizing) and tumour regression may occur in case of a favourable response (downstaging). Downsizing and downstaging together contribute to an increased ratio of resectability and, with it, a higher chance of organ preserving surgery, which considerably improves the later quality of life of patients.

An argument for the preoperative treatment is that tissues have better blood and oxygen supply before the planned surgery, which improves their sensitivity to the treatment. At the same time, the regeneration ability is also better compared with the postoperative adjuvant therapy. The beneficial effect of neoadjuvant therapy on survival has been shown previously (16).

During our research, the effects of modifying the neoadjuvant oncological treatment protocol on tumour regression, the results of the surgical–oncological interventions, the number of lymph nodes removed, the resection margins and the surgical complications, as well as the side effect profile of the treatments, were evaluated.

## Material and methods

Review Board of Human Investigations at the University of Szeged, Hungary, approval number: 117/2020-SZTE.

Neoadjuvant chemotherapy: Previously, patients received a combination of epirubicin, cisplatin and fluorouracil (ECF), and then they were switched to the combination of epirubicin, cisplatin, and capecitabine (ECX). During the ECF/ECX treatment, epirubicin 5 mg/m^2^ (on Day 1), cisplatin 60 mg/m^2^ (on Day 1), and 5-FU 200 mg/m^2^ (or capecitabine 1,250 mg/m^2^ orally, divided into two doses between Day 1 and Day 21) were administered every three weeks. The new pre-treatment was the FLOT therapy, the components and dosages of which were as follows:.

FLOT therapy:
F: 5-FU 2,600 mg/m^2^ in 24-hour IV infusion on Day 1
L: leucovorin 200 mg/m^2^ in IV infusion on Day 1
O: oxaliplatin 85 mg/m^2^ in IV infusion on Day 1
T: docetaxel 50 mg/m^2^ in IV infusion on Day 1
repeated every two weeks.

### Study period

Data from gastric and cardia tumour patients receiving neoadjuvant therapy and then surgery at the Department of Surgery of the University of Szeged between 31 Dec 2008 and 31 Oct 2022 were evaluated during the research.

### Patient inclusion criteria

The criteria for inclusion included disease resectability and an initial stage of at least T_2_ (advanced), without distant metastases and with lymph node positivity (cN+).

### Patient exclusion criteria

Exclusion criteria included potentially irresectable tumors with distant metastasis and patient unfit for neoadjuvant FLOT chemotherapy.

### Patient demographics

Data from a total of 88 patients (35 females and 53 males) were evaluated. The ECF/ECX group (Group 1, *n* = 36) included 36 patients, whereas FLOT was administered to 52 patients (Group 2, *n* = 52).

The mean age of patients and its distribution by gender and treatment group were assessed. In addition, BMI and ASA of the patients were analysed by treatment group.

### Investigations

As part of routine investigations, patients were subjected to oesophago-gastroscopy, sample collection for histology, oncological staging, laboratory tests, and consultation with an anaesthetist. Additional cardiac risk assessment was also performed (ECG, cardiac ultrasound), if it was required.

The T stage was determined using a CT/MRI scan and/or endosonography. No second, restaging MRI scan was performed after the different neoadjuvant oncological treatments.

The ratio of cases with endosonography performed increased over time.

Tumour marker measurements: CEA and CA 19-9 levels were determined in the laboratory before the start of treatment.

### Decision by the tumour board

Decision on neoadjuvant treatment was made in each case by the multidisciplinary (oncology) tumour board. Provided that the patient accepted decision on pre-treatment, neoadjuvant chemotherapy could be initiated. Patients with metastatic or irresectable disease were excluded from this study.

### Timing of surgery

The time to surgery (days) was evaluated both in patients receiving the ECF/ECX treatment protocol and in those subjected to the neoadjuvant FLOT therapy.

The type of further surgical treatment was also determined or much affected by the location of the disease.

### Surgical treatment

During the two different neoadjuvant treatments, surgeries were performed by the same three surgeons experienced in gastric and esophageal surgery. Both open and laparoscopic procedures were performed, using standardised surgical techniques. The technique used was decided by the operating surgeon in each case. Total gastrectomy was carried out with open surgical technique only. Both open surgery and a minimally invasive technique (laparoscopic abdominal phase and thoracoscopy-assisted thoracic phase) were used for cardia resections. Upper midline laparotomy was used for total gastrectomies. Standard D2 lymphadenectomy was performed during both total gastrectomies and cardia resections. During the gastrectomies, a nasojejunal tube was inserted through the anastomosis, all the way below the level of the distal anastomosis, and early enteral feeding was started through it on postoperative day 2. The proximal anastomosis was an end to side esophagojejunal type, made with size 25 circular stapler. The end of the small afferent loop was closed with a linear stapler. The distant anastomosis was handsewn in one layer in the jejunum, 40 cm from the proximal anastomosis. These were end to side anastomoses. In case of cardia resections, a nasogastric tube was inserted into the gastric conduit through the esophagogastric anastomosis, with the purpose of decompression. During the abdominal phase, a jejunal catheter was also inserted to start early enteral feeding. For the minimally invasive cardia resections, the abdominal phase was performed laparoscopically. Main steps of the procedure: preparation of the greater curvature, gastric conduit formation using endoscopic staplers, complete lymphadenectomy, transhiatal mobilisation of the distal third of the esophagus, jejunal catheter insertion, abdominal drainage. After changing patient position, the thoracoscopy-assisted thoracic phase was performed. The proximal anastomosis was an end to side esophagogastric type, made with size 25 circular stapler. The end of the small gastric afferent loop was closed with a linear stapler. The specimen was removed using mini-thoracotomy. A nasogastric tube was inserted into the gastric conduit through the anastomosis, and two chest drains were left in place.

### Follow-up

Patients were surgically followed up 1 week, 1 month and 1 year after being discharged. The mean follow-up of operable patients was 26 months. At the same time, patients were receiving continuous oncological follow-up and care, and their follow-up is still ongoing to this day. Oncological follow-up is done according to the international protocols.

### Studied parameters

1. Side effect profile analysis of oncological treatments:
  The different side effects of the two chemotherapies and their severity were assessed.
2. Comparison of CT images and pathological regression:
  We analysed how informative the CT scan performed after the neoadjuvant oncological treatment was, and how much it could determine the level of tumour regression. The analysis involved comparing the findings from the second CT scan with the TRG determined during the pathological assessment, and checking the level of correlation between the results.
3. Timing of surgery during the two treatment periods:
  The time from the different treatment methods to the surgeries was analysed.
4. Distribution of the surgical techniques in the two oncological periods:
  The ratio of minimally invasive to open surgeries was assessed in the periods corresponding to the two oncological protocols.
5. Assessment of perioperative complications:
  Results were also compared by the neoadjuvant treatment and the surgical technique used. The length of hospital stay (days), the rate of suture failure, as well as the incidence of impaired wound healing and wound suppuration were also assessed. Suture failure was established if contrast leak was revealed by the swallow test performed using a water-soluble contrast agent on postoperative day 7.
6. Pathological evaluation methods of the efficacy of the oncological treatment:
  6.1 TRG analysis: The efficiency of the neoadjuvant oncological treatment was confirmed with a pathological processing of the specimen obtained during the post-treatment surgery. In both periods, laparoscopic surgeries were compared with laparoscopic surgeries and open surgeries were compared with open surgeries for the studied parameters. The TRG–Mandard score was the most important studied parameter.
  6.2 Proximal resection margin: It was assessed if there was any difference in the distance from the tumour to the proximal resection margin between the oncological protocols and between the different surgical techniques.
  6.3 Lymph node status: The two oncological protocols and the two surgery types were assessed, respectively, for any difference in the number of regional lymph nodes removed.

### Statistics

Statistical analyses were performed with STATA 16 program (StataCorp, College Station, TX 77845, United States). Continuous variables were checked for normality using the Shapiro–Wilk test. Two-sample *t*-test and one-way ANOVA were used to compare the means of two or more samples, respectively. If the distribution was not normal, then the Wilcoxon rank sum test or the Kruskal–Wallis test was applied. The proportions were analysed using the chi-squared test and Fisher's exact test. Henceforward, significant results are indicated using asterisks (*p ≤ 0.05; **p ≤ 0.01; ***p ≤ 0.001). The abbreviation “NS” will be used for non-significant *p*-values.

## Results

### Patient demographics

Data from a total of 88 patients were evaluated in our research, with 36 patients in the ECF/ECX group (Group 1, *n* = 36) and 52 patients receiving FLOT (Group 2, *n* = 52).

There were 35 female and 53 male patients. Mean age was 61.65 years in women and 62.35 years in men. There was no significant difference in gender distribution between the ECF/ECX group (Group 1) and the FLOT group (Group 2). (Fisher's exact test; *p* = 0.659) As to mean age, there was a significant difference between the ECF/ECX group (Group 1) and the FLOT group (Group 2) (Student's *t*-test; *p* = 0.0435).

The mean body mass index of the patients in the two different neoadjuvant treatment groups was almost the same (25.50 in the ECF/ECX group vs. 25.90 in the FLOT group). There was no significant difference in the mean BMI between the ECF/ECX group (Group 1) and the FLOT group (Group 2). (Student's *t*-test *p* = 0.6903) (Table 1).

**Table 1:** Mean BMI, mean age, and gender distribution by oncological protocol (ECF/ECX and FLOT).

Type of neoadjuvant therapy	ECF/ECX (*n* = 36)		FLOT (*n* = 52)		*p*-value
Age (with SD)	59.75 ± 11.59		64.48 ± 9.95		*p* = 0.0435 (Student's *t*-test)
Sex (no)	Female: 13	Male: 23	Female: 22	Male: 30	*p* = 0.659 (Fischer exact-test)
BMI (with SD)	25.45 ± 4.97		25.95 ± 5,07		*p* = 0.6903 (Student's t-test)

There was no significant difference between the ECF/ECX group (Group 1) and the FLOT group (Group 2) in ASA classification, including the ASA 1, ASA 2, and ASA 3 classes each (Fisher's exact test) (Table 2).

**Table 2:** ASA classification of patients by treatment group (ECF/ECX and FLOT).

ASA	ECF/ECX, *n* = 36	FLOT, *n* = 52	*p-*value (Fisher exact test)
1	5/36 13.89%	6/52 11.54%	NS
2	21/36 58.33%	25/52 48.07%	NS
3	10/36 27.78%	21/52 40.38%	NS
4	–	–	–
5	–	–	–

### Tumour locations

**Figure 1:**
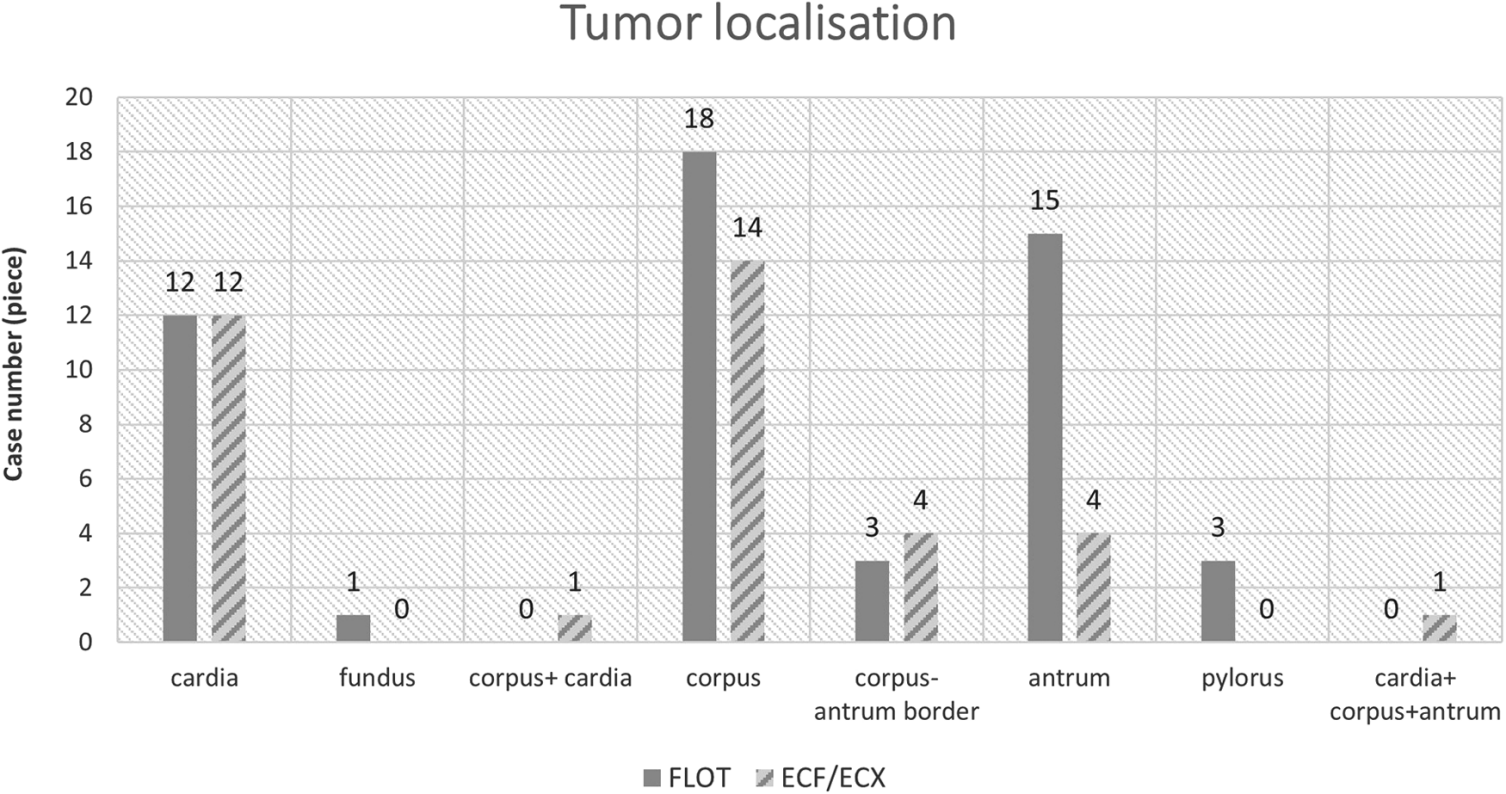
Location of gastric and gastric cardia tumours by treatment group (ECF/ECX and FLOT).

**Figure 2:**
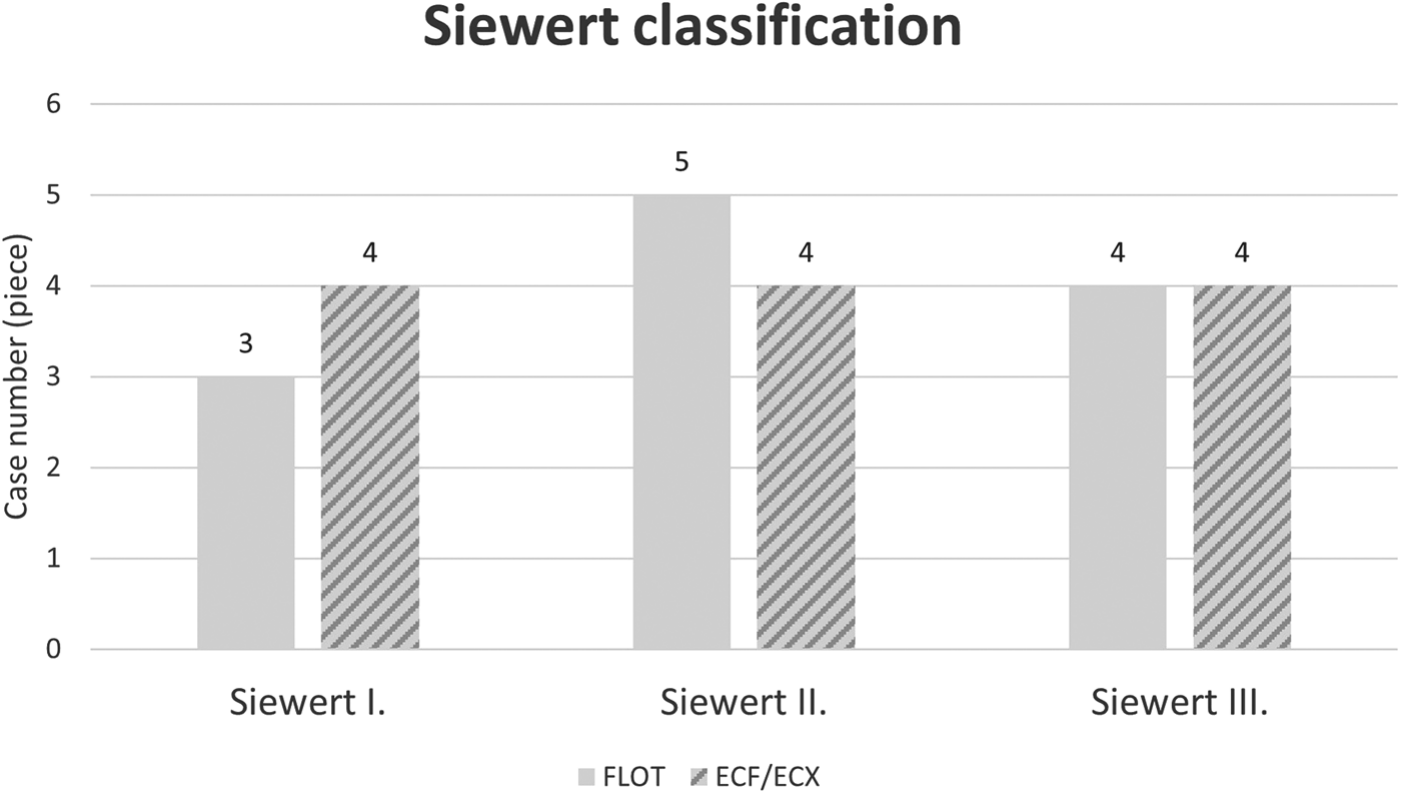
Location of cardia tumours according to the Siewert classification by oncological treatment group (ECF/ECX and FLOT).

The most frequent tumour location was the middle third of the stomach (in 32 out of 88 cases, 36.36%), and the tumours most often showed concentric, “napkin ring”-like spreading (Figures 1, 2).

### Radiological assessment results

A CT scan was performed in all 88 cases and a lesion could be diagnosed already with the CT scan in 66 cases (75.00%). A second CT scan after the completion of the treatment was performed in 66 cases (75.00%). An MRI scan was performed before the start of the treatment, during the previous oncological therapy (ECF/ECX) in 5.56% of the cases, whereas it was performed during the modified oncological therapy (FLOT) in 17.30% of the cases. No second MRI scan was performed after the different neoadjuvant oncological therapies. Certainly, this ratio has improved considerably in accordance with the international recommendations. The ratio of cases with endosonography performed increased over the study period. Endosonography was performed in 38.90% of the cases before the initiation of the previous oncological treatment protocol and in 59.60% of the cases before the modified oncological treatment (FLOT) (Table 3).

**Table 3:** Imaging examinations performed by treatment group (ECF/ECX and FLOT).

Diagnostic procedure	ECF/ECX (*n* = 36)	FLOT (*n* = 52)
Endoscopy	29/36 80.56%	39/52 75.00%
Endosonography	14/36 38.89%	32/52 61.54%
CT	36/36 100.00%	52/52 100.00%
MRI	2/36 5.56%	9/52 17.31%

The laboratory measurement of CEA and CA 19-9 levels did not prove to be informative because of the too high SD values. In accordance with the literature, these markers have an emphasised role rather during follow-up.

Based on radiological imaging methods, patients usually had an N_0_, N_1_ or N2 lymph node involvement, with only 4 patients having a stage N_3_ gastric tumour included in the study. In case of metastasis, the radiological picture of the distant metastasis was not typical and, therefore, the diagnosis of a metastasis could not be confirmed safely.

As to the initial T stage (including T_1_, T_2_, T_3_, and T_4_ each), there was no significant difference between the ECF/ECX group (Group 1) and the FLOT group (Group 2) (Fisher’ exact test; *p* = 0.082).

The difference in the initial N stage between the ECF/ECX group (Group 1) and the FLOT group (Group 2) was not significant (Fisher's exact test; *p* = 0.603).

**Figure 3:**
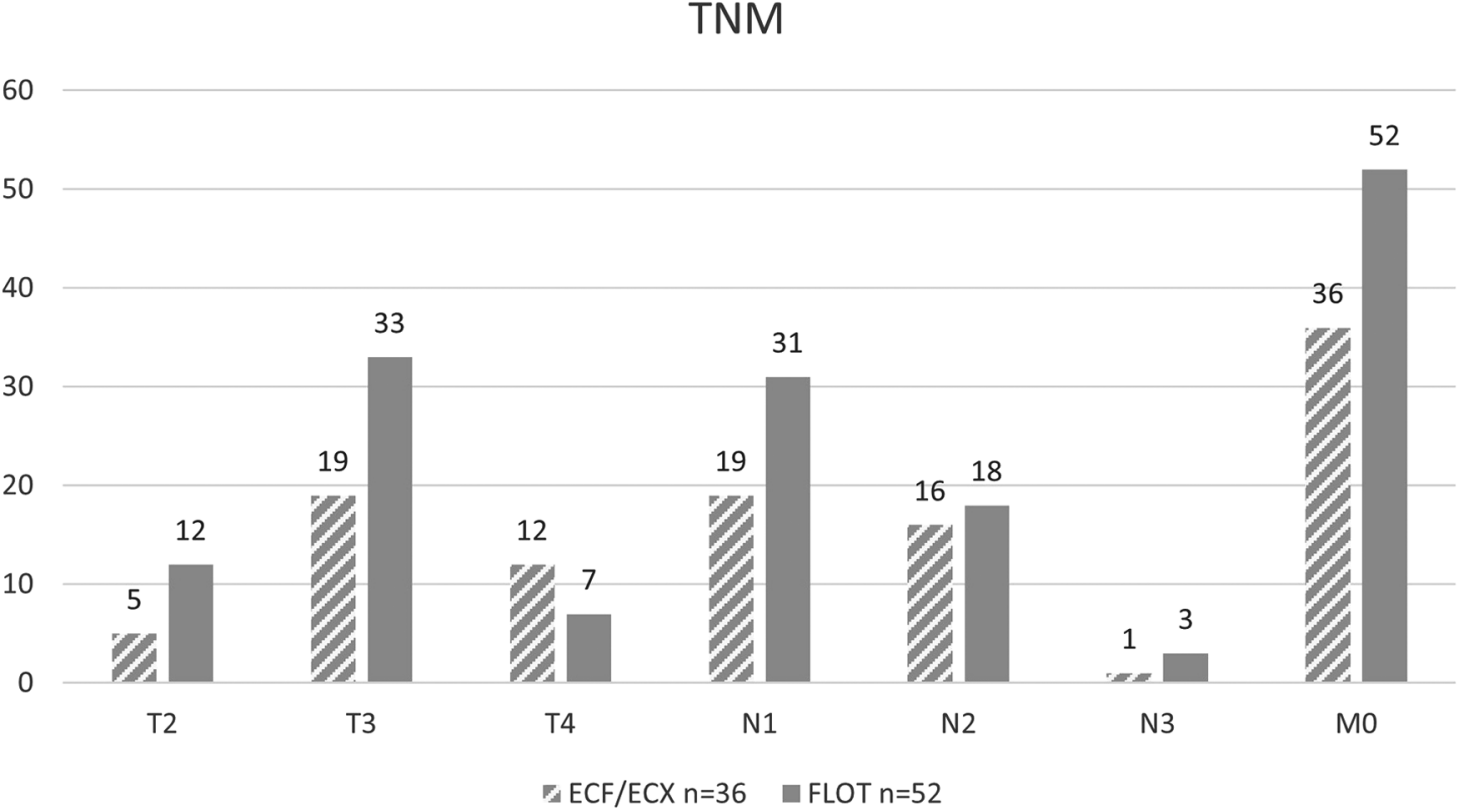
Initial TNM stage of the patients by oncological protocol (ECF/ECX and FLOT).

As to the initial M stage, there were no cases with distant metastasis in either the ECF/ECX group (Group 1) or the FLOT group (Group 2) (Figure 3).

#### Side effect profile analysis

a) Diarrhoea: A change in bowel habits is a very common side effect during chemotherapy. During ECF/ECX therapy, Grade 1 diarrhoea occurred in 2 out of the 36 cases (5.55%); Grade 2 diarrhoea was developed in 1 out of the 36 cases (2.78%); and Grade 3 diarrhoea was not reported. During the FLOT therapy, Grade 1 diarrhoea occurred in 10 out of the 52 cases (19.23%); Grade 2 diarrhoea was developed in 3 out of the 52 cases (5.77%); and Grade 3 diarrhoea was reported in 1 out of the 52 cases (1.92%). There was a significant difference between the ECF/ECX group (Group 1) and the FLOT group (Group 2) in the rate of diarrhoea (Grade 1, 2, and 3 diarrhoea each) (Fisher's exact test; *p* = 0.006).
b) Weight loss: A small number of patients showed minimal weight loss during the intravenous chemotherapies. Whereas Grade 1 weight loss was reported in 2 out of the 36 cases (5.55%) in the ECF/ECX group, it occurred in 4 out of the 52 cases (7.69%) in the FLOT group. With regard to the side effect of weight loss, there was no significant difference between the ECF/ECX and the FLOT treatments (Fisher's exact test; *p* = 1.000).
c) Nausea: The leading symptom of intravenous chemotherapies. Nausea and vomiting were predominant in this study as well, occurring in both study periods. During the ECF/ECX treatment, Grade 1 and Grade 2 nausea occurred in 17 (47.22%) and 2 (5.55%) of the 36 cases, respectively, whereas during the FLOT treatment, the rates of Grade 1 and Grade 2 nausea were 11 (21.15%) and 2 (3.85%) of the 52 cases, respectively. There was no significant difference between the ECF/ECX and FLOT groups in Grade 1 and Grade 2 nausea and vomiting (Fisher's exact test; *p* = 0.192).
d) Neutropenia: No significant difference was demonstrated between the two oncological protocols in the production of cellular blood components. Neutropenia was slightly more common during the ECF/ECX treatment, which was associated with Grade 1, Grade 2 and Grade 3 neutropenia in 2 (5.55%), 1 (2.78%) and 1 (2.78%) of the 36 cases, respectively, whereas in the FLOT group, Grade 1 neutropenia occurred in 3 out of the 52 cases (5.77%). No Grade 2 or Grade 3 neutropenia was observed during FLOT treatment. Regarding neutropenia (including Grade 1, Grade 2, and Grade 3 cases), there was no significant difference between the ECF/ECX and FLOT treatments (Fisher's exact test; *p* = 0.294).
e) Anaemia: There was a significant difference in the rate of treatment-emergent anaemia. During the pre-treatment with ECF/ECX, patients developed Grade 1 and Grade 2 anaemia in 3 (8.33%) and 2 (5.56%) of the 36 cases, respectively. The FLOT therapy was not associated with Grade 1 anaemia but Grade 2 anaemia was observed in 2 out of the 52 cases (3.85%). (Cut-off values in males: haematocrit: 0.39%; haemoglobin: 133 g/L; in females: haematocrit: 0.36%, haemoglobin: 118 g/L.) There was a significant difference in the rate of anaemia between the ECF/ECX and FLOT treatments (Fisher's exact test; *p* = 0.036).
f) Peripheral neuropathy: The ECF/ECX treatment was not associated with Grade 1 peripheral neuropathy but Grade 2 peripheral neuropathy occurred in 1 out of the 36 cases (2.78%); with the FLOT pre-treatment, Grade 1 and Grade 2 peripheral neuropathy was developed in 10 (19.23%) and 1 (1.92%) of the 52 cases, respectively. The difference between the ECF/ECX and FLOT treatments in the rate of peripheral neuropathy was not significant (Fisher's exact test; *p* = 0.192).
g) Fever: During the pre-treatment with ECF/ECX, it occurred in 1 out of the 36 cases (2.78%), whereas in the FLOT group, it was reported in 1 out of the 52 cases (1.92%). The difference between the ECF/ECX and FLOT treatments in the rate of fever was not significant (Fisher's exact test; *p* = 1.000).

No other special, treatment-related complications were observed during either the ECF/ECX or the FLOT treatment (Table 5).

#### Comparison of CT images and pathological regression

In our study, clear improvement, regression was established in case of TRG 1-2. TRG 3-4 was deemed minimal improvement or unchanged status. TRG 5 meant no improvement. Based on the results, the tumour response, considering regression, found during the second, restaging CT scan correlated with the TRG in only 48.48% of the cases. Tumour response to the neoadjuvant oncological treatment was qualified, based on the follow-up CT scan, as better or worse (considering TRG 5 as progression only) than the result from the postoperative pathological assessment in 25.00% and 7.14% of the cases, respectively (Table 4).

**Table 4:** Side effects of the neoadjuvant treatments in the ECF/ECX and FLOT groups.

Type of side effect	ECF/ECX, *n* = 36	FLOT, *n* = 52	*p*-value (Fisher's exact test)
Vomiting, Grade 1	17/36 47.22%	11/52 21.15%	*p* = 0.192
Vomiting, Grade 2	2/36 5.55%	2/52 3.85%	
Anaemia, Grade 2	3/36 8.33%	2/52 3.58%	*p* = 0.036
Anaemia, Grade 3	2/36 5.55%	0/52	
Diarrhoea, Grade 1	2/36 5.55%	10/52 19.23%	*p* = 0.006
Diarrhoea, Grade 2	1/36 2.78%	3/52 5.77%	
Diarrhoea, Grade 3	0/36	1/52 1.92%	
Neutropenia, Grade 1	2/36 5.55%	3/52 5.77%	*p* = 0.294
Neutropenia, Grade 2	1/36 2.78%	0/52	
Neutropenia, Grade 3	1/36 2.78%	0/52	
Peripheral neuropathy, Grade 1	0/36	10/52 19.23%	*p* = 0.192
Peripheral neuropathy, Grade 2	1/36 2.78%	1/52 1.92%	
Fever	1/36 2.78%	1/52 1.92%	*p* = 1.000
Weight loss	2/36 5.55%	4/52 7.69%	*p* = 1.000

These study results confirm the well-known fact that CT scans are not suitable for assessing the degree of tumour response to the oncological neoadjuvant treatment.

#### Timing of surgery

In the two studied periods, surgery was performed a mean 6.12 weeks and a mean 5.82 weeks after the ECF/ECX and FLOT treatments, respectively. There was no significant difference in the time from the two different oncological treatments to the surgery.

#### Distribution of the surgical techniques in the two oncological periods

Out of the 88 patients who went through surgery, 23 patients received no curative surgery because of complete technical and oncological inoperability (locally advanced status, carcinosis, spreading to adjacent organs).

**Figure 4:**
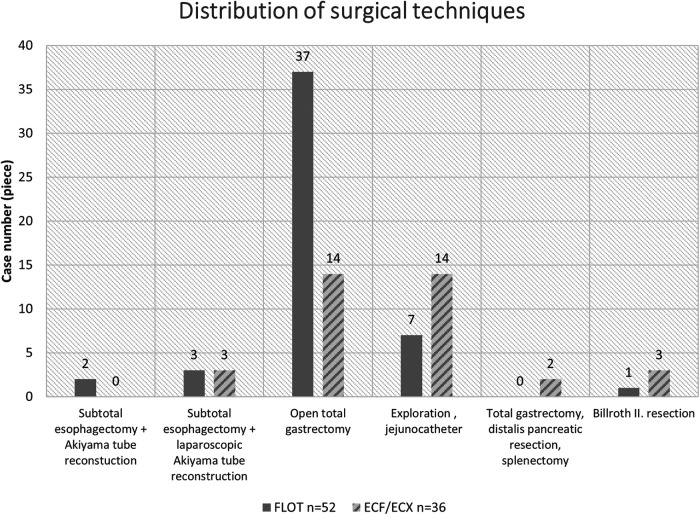
Distribution of the surgical techniques in the two oncological periods.

Based on our results, the rate of laparoscopic procedures was 6.65% higher after the previous ECF/ECX neoadjuvant treatment (3/22) than following the FLOT pre-treatment (3/43) (Figure 4).

#### Assessment of perioperative complications

##### Anastomotic failure

When analysing complications, considering that our subject is esophageal surgery, the assessment of anastomotic failure has a special importance, not only with regard to the different neoadjuvant protocols but also to the two types of surgical intervention. The swallow test performed with a water-soluble contrast agent on the seventh postoperative day revealed some degree of contrast leak or a sign of anastomotic failure in 2 (9.09%) of the 22 cases in the ECF/ECX group and in 5 (11.63%) of the 43 cases in the FLOT group. As to anastomotic failure following a curative surgery, there was no significant difference between the ECF/ECX and FLOT treatments (Fisher's exact test; *p* = 0.697).

##### Repeat surgery, impaired wound healing

Immediate repeat surgery was required in one case among those with ECF/ECX pre-treatment, following an open surgery in a patient on dual anticoagulation therapy, because of diffuse bleeding; local haemostasis, hemostyptics, lavage, and drainage were given.

Wound suppuration as a complication occurred, overall, regardless of the type of surgery and the surgical technique used, in 8 out of the 36 cases in the ECF/ECX group (22.22%). It was reported in 8 (15.38%) of the 52 cases following the FLOT treatment. All cases of wound suppuration resolved to conservative therapy (local wound treatment, antibiotics), repeat operation was not required in either group. There was no significant difference in impaired wound healing between the ECF/ECX and FLOT treatments (Fisher's exact test; *p* = 0.301).

##### Hospital stay

The mean length of hospital stay was 13 days in both the ECF/ECX group and the FLOT group.

#### Efficacy results of the oncological treatment

##### 3.2.6.1. Tumour regression grade analysis

**Figure 5:**
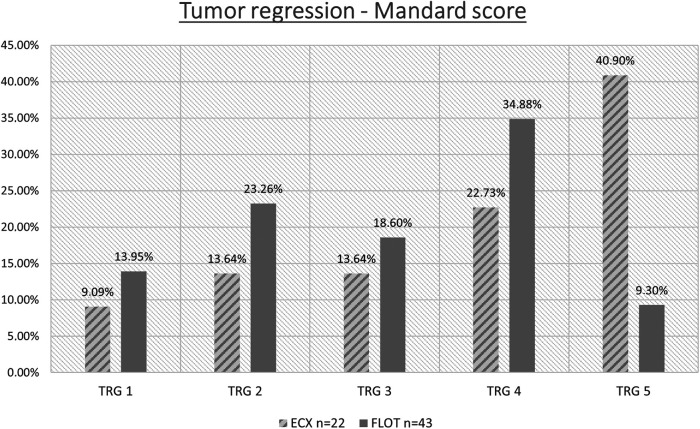
TRG values in the ECF/ECX and FLOT groups.

Data from the 65 operable patients were classified according to the 5 grades corresponding to the Mandard score, by oncological pre-treatment protocol. Complete tumour regression (TRG 1) was reported in a total of 8 cases, out of which 6 were the result of the modified neoadjuvant FLOT chemotherapy. Complete tumour regression occurred in 9.09% and 13.95% of the cases in the ECF/ECX and FLOT groups, respectively. (Figure 5 and Table 6) The modified oncological treatment (FLOT) resulted in a significantly higher rate of complete tumour regression (TRG 1) (Fisher's exact test; *p* = 0.042).

**Table 5:** Clinical response based on CT/MRI findings in the ECF/ECX and FLOT groups.

Clinical response based on CT/MRI findings	ECF/ECX (*n* = 36)	FLOT (*n* = 52)
Not rated	7/36 19.44%	13/52 25.00%
Regression	15/36 41.67%	18/52 34.62%
Unchanged	10/36 27.78%	10/52 19.23%
Progression	4/36 11.11%	11/52 21.15%

**Table 6:** TRG values in the ECF/ECX and FLOT groups. .

	ECF/ECX, *n* = 22	FLOT, *n* = 43	*p*-value (Fisher's exact test)
TRG 1	2/22 9.09%	6/43 13.95%	*p* = 0.042
TRG 2	3/22 13.63%	10/43 23.26%	
TRG 3	3/22 13.63%	8/43 18.06%	
TRG 4	5/22 22.73%	15/43 34.88%	
TRG 5	9/22 40.90%	4/43 9.30%	

##### Proximal resection margin

Following open total gastrectomies, proximal resection margins showed a distance of 66.11 mm (ECF/ECX) vs. 54.36 mm (FLOT). There was no significant difference between the two oncological pre-treatments in the proximal resection margin following an open surgery (Mann–Whitney *U* test; *p* = 0.9501). The same was true for laparoscopic procedures, where there was no significant difference either (Mann–Whitney *U* test; *p* = 0.500).

Overall, regardless of the surgical technique used, R1 resection was achieved in 3 (13.64%) of the 22 cases in the ECX/ECF group and in 4 (9.30%) of the 43 cases in the FLOT group. The difference between the ECF/ECX and FLOT treatments in the achievement of R0 and R1 resection was borderline significant (Fisher's exact test; *p* = 0.055) (Table 7).

**Table 7:** Resection margins by oncological pre-treatment protocol.

	ECF/ECX, *n* = 22	FLOT, *n* = 43	*p*-value (Fisher's exact test)
R0	19	39	*p* = 0.055
R1	3	4	

##### Number of removed lymph nodes

The mean total number of lymph nodes removed during the surgeries was 20.13 and 24.69 in the ECF/ECX and FLOT groups, respectively. The number of lymph nodes removed was further analysed by surgical technique. After pre-treatment with ECF/ECX, the mean number of lymph nodes removed was 19.63 and 23.33 during open surgeries and laparoscopic procedures, respectively. Following FLOT pre-treatment, the mean number of lymph nodes removed was 25.42 and 18.33 during open surgeries and laparoscopic procedures, respectively. There was no significant difference between the two oncological pre-treatments in the mean total number of lymph nodes removed. Mean number of positive lymphnodes were 5 in ECF/ECX group and 1,35 in FLOT group. As to the total number of positive lymph nodes removed, there was a significant difference between the two oncological pre-treatments (Mann–Whitney *U* test; *p* = 0.0267).

#### Tumour marker measurement results

The laboratory measurement of CEA and CA 19-9 levels did not prove to be informative because of the high SD values. In accordance with the literature, these markers have an emphasised role rather during follow-up.

## Discussion

The management of gastric tumours and tumours in the distal third of the oesophagus requires complex care, the main pillars of which are proper diagnostics, an oncological therapy continuously being advanced with new drugs and procedures, and a properly planned and performed surgical treatment (15). It is important to be able to support the efficiency of modified oncological treatments also with real-world results. Choosing the correct treatment strategy for gastric and cardia tumours, as well as tumours located in the distal oesophagus, warrants a multidisciplinary (tumour board) decision, and great experience and proficiency are required on the part of the surgeon (16). Today, relevant quality assurance principles can only be fulfilled with the regulated, regular operation of tumour boards.

Over the past decade, there has been a considerable change in approach, treatment strategies have been transformed, and classifications that are new from many aspects have been developed for oesophageal, cardia and gastric tumours. It suffices to mention the new classifications that appeared in the 7th edition of TNM and categorise positive lymph nodes (17, 18). The changes were needed because of the different prognostic groups based on the number of metastatic lymph nodes (19, 20).

TNM 8 also brought novelties in this field; the classic Siewert type I and II tumours mentioned previously are now considered oesophageal tumours and, correspondingly, their management follows the therapeutic algorithms used for oesophageal tumours (21, 22).

Neoadjuvant therapy has been part of the treatment for patients with advanced gastric, GEJ and oesophageal tumours for more than two decades now. Its justification is unquestionable, and any change in the treatment methods has a considerable impact also on surgery, among others (23).

However the type of neoadjuvant regimens differ by geographic locations of these patients. For patients with locally advanced esophageal and GEJ adenocarcinoma, one of the most commonly used treatment option consists of neoadjuvant chemoradiation with carboplatin/paclitaxel prior to surgery (CROSS study) (24).

Interestingly, while modifying the neoadjuvant treatment protocols, the addition of oxaliplatin (FLOT) resulted in a higher rate of pCR—as expected—, but neither improved survival or increased locoregional control can be reported yet (25).

There have been attempts at intensifying the FLOT treatment by administering 6 cycles of therapy instead of the usual 4 cycles. There was no significant difference in the number of perioperative complications. A higher rate of R0 resections and an improved ratio of metastatic/normal lymph nodes may be the advantages of the prolonged treatment but the “standard” is still the 4 cycles of treatment (26).

Further studies were conducted, among others, with a combination of the FLOT treatment, when spartalizumab was added to the four-drug treatment in the Phase 2 GASPAR study (27).

Previously as the combination of 5-fluorouracil with oxaliplatin or cisplatin were studied with lower toxicity compared to original FLOT protocol(FLAGS trial) (28).

In addition, the combination of FLOT and HIPEC was also assessed in multicentre randomised studies. Early recurrence with carcinosis and markedly poor prognosis is common after successful R0 resection of advanced tumours. In case of diffuse gastric and GEJ II–III tumours, patients also received intraoperative intaperitoneal cisplatin in one of the arms. The first patient was enrolled in 2021 (29).

During our study, not only did we assess the effects of the two different neoadjuvant oncological treatments on patients with gastric and cardia tumours, but we also evaluated the results by the type of surgery, where, aiming at complete homogeneity, results from open surgeries were compared only with those from open surgeries, and laparoscopic results were compared only with data from patients subjected to laparoscopy. The more favourable response of the tumour to the oncological treatment following FLOT therapy was confirmed in our patients based on the Mandard score. The better efficiency and effectiveness of the new combined chemotherapy, compared with the previous ECF/ECX treatment, can be measured well and in a standardised way based on TRG. The assessments clearly show that FLOT has favourable side effect profile and, what is more, that certain life-threatening side effects—occurring with ECF/ECX—are almost completely absent.

Based on the number of lymph nodes removed and the distances from the resection margins, the modification of the neoadjuvant treatment protocol did not increase “oncological radicality”. Beyond its biological impact, the change in the oncological therapy also had an effect on the surgical treatment. Although this difference did not prove to be significant, it contributed considerably to an improvement in the ratio of oncological and technical operability. Certainly, there are still undecided questions such as that about the type of surgery for patients with a classic Siewert type II adenocarcinoma. Previously, tumours with a Siewert II location were considered a separate “entity” where a more aggressive behaviour resulted in a higher rate of recurrence than in the other two classes. Accordingly, surgical procedures as radical as possible were insisted on for such tumours (30). Statements by the two opposing parties can be found in the study results from the FREGAT working group and the CARDIA trial (31, 32). The question is whether adenocarcinomas in a Siewert II location should be treated with a) transhiatal extended total gastrectomy performed using a minimally invasive method, with complete D2 lymphadenectomy, or b) distal oesophageal resection and resection of the superior pole of the stomach (SPO) with gastric sleeve formation and, among others, mediastinal lymphadenectomy, and intrathoracic anastomosis (33, 34). An argument against transhiatal total gastrectomy is the high rate of positive oral resection margin (R1), which was 12% in the total gastrectomy group and 5.9% in the SPO group. According to the study conducted at 21 centres in France, the mean survival was significantly longer in the total gastrectomy group (46 months in the TG group vs. 27 months in the SPO group) (FREGAT working group). The opposing party believes in transthoracic oesophageal resection completed with mediastinal lymphadenectomy. The emphasis is on mediastinal lymphadenectomy, since Siewert II adenocarcinomas—naturally, depending on their stage—may be associated with up to 10% of positive mediastinal lymph nodes. According to their investigations, total gastrectomies are associated with a higher rate of recurrence, a lower ratio of disease-free survival due to the positive, metastatic lymph nodes left in the mediastinum. Our position, which is based on our own results, agrees with the opinion and partial results of the CARDIA trial.

As to the surgery of malignant cardia tumours, thoracoscopy-assisted minimally invasive laparoscopy has been the “gold standard” treatment for almost a decade now (35–38). After the results from the TIME trial, the question in the surgery of cardia tumours is no longer whether minimally invasive procedures are justified but what method or technique should be used during them. Compared with open surgeries (39, 40), minimally invasive procedures are associated with less blood loss, less need for postoperative analgesia, and a considerably lower rate of pulmonary complications.

Nowadays minimally invasive surgery offers better survival and improved short-term postoperative outcomes in gastric and GEJ cancers compared to classic open procedures (41).

Patients may be mobilised earlier and the result is aesthetically better. The length of hospital stay can be decreased significantly. Within minimally invasive procedures, the results of robotic surgery are gradually improving, and the outcomes reported by expert centres are highly convincing. Numerous comparative studies have published their results (42).

The safe and oncologically equivalent use of robotic surgery is unquestionable but the results from additional ongoing, prospective, randomised, multicentre studies will help further analysis (43, 44).

Continuing with the analysis of the results from the two different pre-treatments, we observed slightly more favourable results overall in the FLOT group regarding passage disorders and wound suppuration among the complications reported during the immediate perioperative period, but these did not reach the level of significance. As to the highly important anastomotic failure, no true, significant difference could be shown between the two pre-treatment methods. The short-term benefits are unquestionable. Besides the favourable side effect profile and the slightly more favourable or at least unchanged perioperative and late postoperative complications, tumours show a considerably more favourable response to the modified oncological pre-treatment. To date, no reliable studies have been conducted to confirm any possible effect on long-term survival. We continue to collect and analyse relevant data.

In conclusion, there was a significantly higher rate of complete tumour regression when advanced gastric and cardia tumours were treated with the new FLOT neoadjuvant chemotherapy. The side effect profile of the new, modified treatment proved to be favourable compared with previous protocols. It can also be said that the modification of the oncological protocol also had an effect on the outcome of surgery, since there was an increase in the number of curative, oncologically correct R0 surgeries following the treatment.

## Data Availability

The raw data supporting the conclusions of this article will be made available by the authors, without undue reservation.
